# Preferential Exhaustion and Loss of Predominant Reservoir in Gut-Associated Lymph Nodes of a SIVmac239-Infected Rhesus Macaque with Terminal AIDS

**DOI:** 10.3390/v18080855

**Published:** 2026-08-04

**Authors:** Cong-Jiao Bai, Yu Zhang, Zhang-Ran Du, Yu-Qiu Wang, Meng-Xue Sun, Liu-Meng Yang, Yong-Tang Zheng, Tian-Zhang Song

**Affiliations:** 1 College of Life Sciences, Anhui Normal University, Wuhu 241000, China; 15198647408@163.com (C.-J.B.); 19016151762@163.com (M.-X.S.); 2 State Key Laboratory of Genetic Evolution & Animal Models, Key Laboratory of Bioactive Peptides of Yunnan Province, National Resource Center for Non-Human Primates, Kunming Institute of Zoology, Chinese Academy of Sciences, Kunming 650223, China; zhangyu223@163.com (Y.Z.); dzr06292458@163.com (Z.-R.D.); w3094247273@163.com (Y.-Q.W.); lmyang@mail.kiz.ac.cn (L.-M.Y.); 3 School of Pharmaceutical Science and Yunnan Key Laboratory of Pharmacology for Natural Products, Kunming Medical University, Kunming 650500, China

**Keywords:** AIDS, SIVmac239, nonhuman primate, gut-associated lymph node, viral reservoir, lymphoid exhaustion

## Abstract

Gut-associated lymph nodes (GALNs) constitute an important anatomical reservoir during early and chronic human immunodeficiency virus (HIV)/simian immunodeficiency virus (SIV) infection, but the fate of this compartment during terminal AIDS remains unclear. This study investigated viral persistence and lymph node injury in a rhesus macaque with long-term SIVmac239 infection and end-stage AIDS, characterized by profound CD4+ T cell depletion (30 cells/μL) and persistent viremia (1192 copies/mL). At necropsy, GALNs, including mesenteric, paracolic, and ileocecal lymph nodes, and non-gut-associated lymph nodes (NGALNs), including hepatic hilar, common iliac, and inguinal lymph nodes, were collected for viral RNA/DNA quantification, histopathology, immunofluorescence, and transcriptomics. SIV DNA was markedly lower in GALNs than in NGALNs, whereas SIV RNA was detectable only in NGALNs. GALNs exhibited extensive fibrotic remodeling, paracortical collapse, severe CD4+ T cell loss across B cell and paracortical regions, and pronounced CD8+ T cell accumulation within B cell zones. Transcriptomic profiling further revealed an exhaustion signature in GALNs, with reduced DNA replication and repair, impaired cell-cycle activity, and suppressed RNA processing, accompanied by activation of innate immune programs and attenuation of adaptive immune responses. These findings indicate that, during terminal AIDS, GALNs no longer serve as the predominant viral reservoir but instead undergo preferential and severe structural and functional exhaustion. This case highlights marked anatomical heterogeneity in lymph node vulnerability during advanced disease and supports a model in which collapse of the gut-associated immune barrier contributes to disease progression toward terminal AIDS.

## 1. Introduction

Combination antiretroviral therapy (cART) durably suppresses human immunodeficiency virus (HIV) replication but does not eradicate infection, primarily due to the persistence of replication-competent virus within anatomical reservoirs. Secondary lymphoid tissues, including the spleen, tonsils, and lymph nodes, are central sites of HIV/simian immunodeficiency virus (SIV) persistence because they contain abundant CD4+ T cells and provide cellular and stromal niches that support viral maintenance [1]. Among these compartments, gut-associated lymph nodes (GALNs) represent a distinct and highly vulnerable reservoir. Unlike superficial or peripheral lymph nodes, GALNs are continuously exposed to dietary antigens and microbial products, creating a state of physiological immune activation that increases CCR5 and CXCR4 expression on CD4+ T cells and enhances susceptibility to HIV/SIV infection [2]. Consequently, viral reservoirs are established earlier in GALNs, reach greater burdens, and persist more readily under cART than those in superficial lymph nodes [3,4].

Chronic HIV/SIV infection progressively remodels lymphoid tissues through fibrosis, architectural disorganization, and CD4+ T cell depletion [1,5]. As this damage advances, the same microenvironment that initially favors viral persistence may lose the cellular and structural capacity required to maintain active viral reservoirs. This uncertainty defines a central question in terminal acquired immunodeficiency syndrome (AIDS): do GALNs remain the predominant viral reservoir after prolonged infection, or does severe structural and functional exhaustion eliminate their capacity to sustain viral persistence? To address this question, GALNs and non-gut-associated lymph nodes (NGALNs) were comparatively analyzed in a rhesus macaque with long-term SIVmac239 infection and progression to AIDS. Viral RNA and DNA burdens, histopathological injury, immune cell distribution, and transcriptomic profiling were integrated to determine the terminal fate of GALNs during advanced disease.

## 2. Materials and Methods

An adult female Chinese rhesus macaque (*Macaca mulatta*) was intravenously inoculated through the posterior tibial vein with 4000 TCID_50_ of SIVmac239 (GenBank: M33262.1) diluted in sterile phosphate-buffered saline (PBS) to a final volume of 2 mL. The viral stock was generated by transfecting CEMx174 cells with the infectious molecular clones p239SpSp5′ and p239SpE3′, which were kindly provided by Dr. Bin Gao (University College London) [6]. This animal was assigned as an untreated control in a larger study evaluating the in vivo efficacy of novel antiviral candidates and therefore received no antiretroviral therapy or other experimental intervention during the 3.5-year infection period. Peripheral blood was collected from the posterior tibial vein at scheduled intervals to longitudinally monitor plasma viral load and CD4/CD8 ratio. The animal was euthanized after reaching predefined humane endpoints, defined as the simultaneous occurrence of at least three of the following criteria: ≥10% body weight loss within 2 weeks, persistent diarrhea for more than 2 weeks, sustained lethargy with daytime recumbency exceeding 2 h for 2 weeks, and peripheral CD4+ T cell count <300 cells/μL persisting for more than 2 weeks. Euthanasia was performed using an overdose of Zoletil 50 (Virbac, Carros, France), followed by transcardial perfusion with precooled PBS. At necropsy, multiple GALNs, comprising mesenteric, paracolic, and ileocecal lymph nodes, and NGALNs, comprising hepatic hilar, common iliac, and inguinal lymph nodes, were collected for downstream analyses. All animal procedures were approved by the Ethics Committee of the Kunming Institute of Zoology, Chinese Academy of Sciences (Approval No.: IACUC-PE-2021-06-001).

Total RNA from plasma was isolated using a High Pure Viral RNA Kit (11858882001, Roche, Basel, Switzerland), whereas total RNA from lymph nodes was extracted using TRIzol Reagent (9109, Takara, Kusatsu, Shiga, Japan). Genomic DNA was purified using a TIANamp Genomic DNA Kit (DP304, TIANGEN, Beijing, China). Viral RNA and DNA were quantified using previously reported primers and probes targeting the SIVmac239 *gag* gene [7]. RNA quantification was performed on a QuantStudio^TM^ 5 Real-Time Polymerase Chain Reaction (PCR) Instrument (Thermo, Waltham, MA, USA) using the Evo M-MLV One-Step RT-qPCR Kit (AG11715, Accurate Biotechnology, Changsha, China), and DNA quantification was performed using the Premix Ex Taq™ (RR390A, Takara, Kusatsu, Shiga, Japan). Standard curves were generated from serial dilutions of the pGEM-4Z-SIVgag357 plasmid. Genomic DNA, extracted from peripheral blood samples, was used for MHC genotyping by PCR with sequence-specific primers (PCR-SSP). Sequence-specific primers were used to detect selected MHC class I alleles/loci, including Mamu-A*01, Mamu-B*03, Mamu-B*08, Mamu-B*17, Mamu-B*1702, and Mamu-B, as well as the MHC class II locus Mamu-DRB (Figure S1A). PCR products were separated by agarose gel electrophoresis, and the presence of expected allele/locus-specific amplification products was used to determine the corresponding MHC genotypes. Flow cytometry was performed as described previously [8]. Absolute T cell counts were determined using fresh EDTA-K2-anticoagulated peripheral blood. The anti-human antibodies used for flow cytometry were cross-reactive with rhesus macaque antigens and included anti-CD3 BV421 (clone SP34-2, BD Biosciences, San Jose, CA, USA), anti-CD8 PE-Cy7 (clone PRA-T8, BD Biosciences, San Jose, CA, USA), and anti-CD4 PerCP/Cy5.5 (clone OKT4, BioLegend, San Diego, CA, USA).

For histopathological analysis, lymph node tissues were fixed in 4% paraformaldehyde (G1101-15ML, Servicebio, Wuhan, China), dehydrated through a graded ethanol series, embedded in paraffin, and sectioned at 4 μm. Tissue sections were stained with hematoxylin–eosin (H&E) and Masson’s trichrome, and brightfield images were captured using a BX53 microscope (Olympus, Tokyo, Japan). Immunofluorescence was performed as described previously [9]. Paraffin sections were incubated with primary antibodies: rabbit anti-CD4 (ab133616, Abcam, Cambridge, UK), rabbit anti-CD8 (ab237709, Abcam, Cambridge, UK), and mouse anti-CD20 (14-0202-82, Invitrogen, Carlsbad, CA, USA). After washing, sections were incubated with secondary antibodies: goat anti-rabbit Alexa Fluor^TM^ 555 (ab150078, Abcam, Cambridge, UK) and goat anti-mouse Alexa Fluor^TM^ 488 (ab150117, Abcam, Cambridge, UK). Nuclei were counterstained with 4′,6-diamidino-2-phenylindole (DAPI; G1012-100ML, Servicebio, Wuhan, China). Immunohistochemical staining was performed using a MaxVision HRP-Polymer anti-Mouse IHC Kit (Maixin, Fuzhou, China). Fluorescence images were acquired using a stimulated emission depletion microscope (STEDYCON, Abberior, Göttingen, Germany).

Bulk RNA sequencing (RNA-seq) was conducted on total RNA extracted from all lymph nodes following established protocols [10]. Briefly, sequencing libraries were generated and sequenced on the Illumina NovaSeq 6000 platform (Illumina, San Diego, CA, USA). Clean reads were aligned to the rhesus macaque reference genome (Mmul_10). Gene expression differences were analyzed using DESeq2, and differentially expressed genes (DEGs) were defined using |log2 fold change| > 1 and *p* < 0.05. Gene set enrichment analysis (GSEA) was performed with the clusterProfiler R package (v4.6.2) using the MSigDB C5 biological process gene set (v7.1; c5.bp.v7.1.symbols). Pathways with adjusted *p* < 0.05 were considered significantly enriched, and the direction of enrichment was determined from the sign of the running enrichment score, with positive and negative values indicating upregulation and downregulation, respectively, in GALNs relative to NGALNs.

## 3. Results

**Figure 1:**
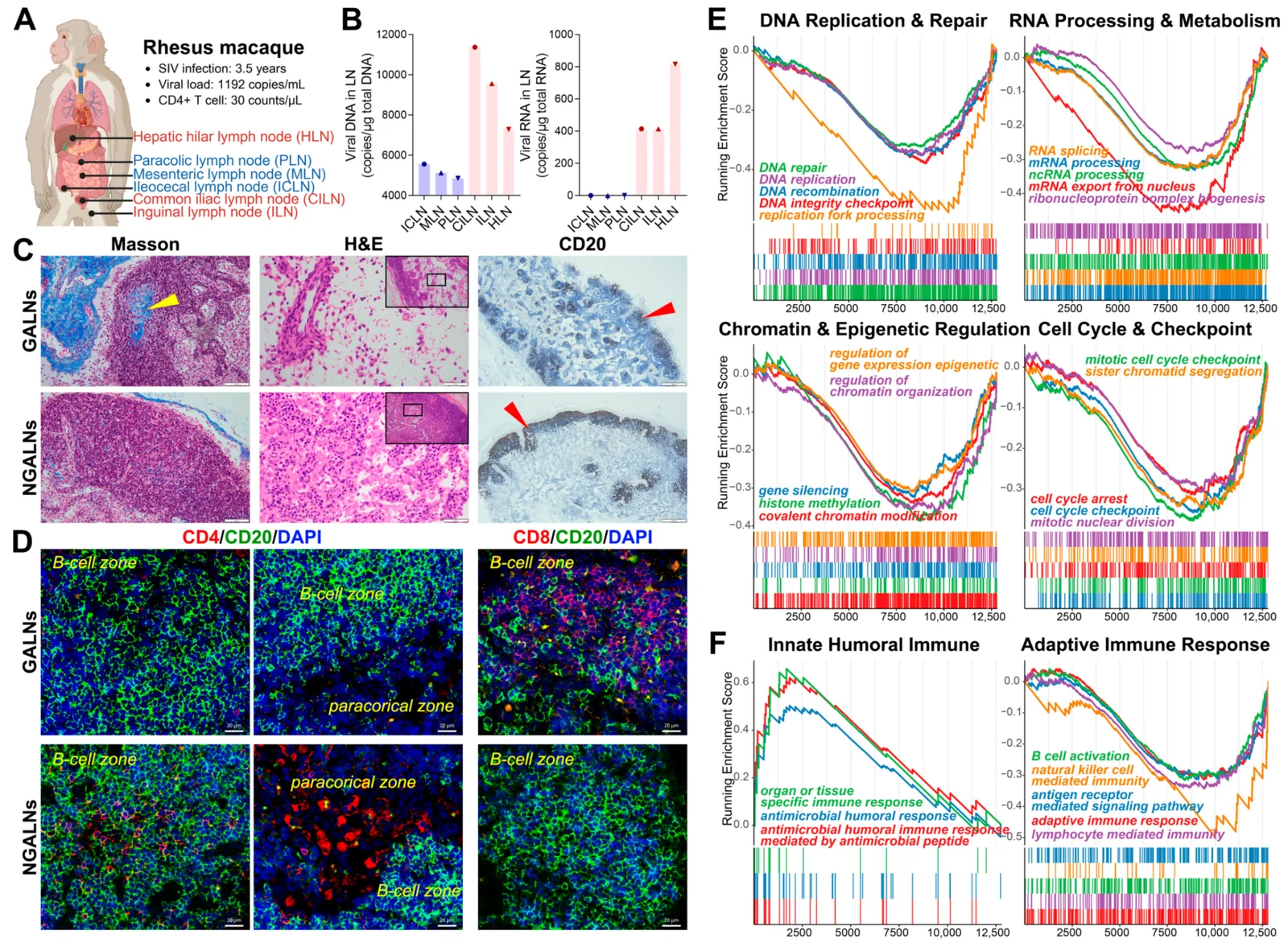
Virological, histopathological, immunological, and transcriptomic differences between gut-associated lymph nodes (GALNs) and non-gut-associated lymph nodes (NGALNs) in a rhesus macaque with terminal AIDS after long-term SIVmac239 infection. (**A**) Anatomical distribution of sampled GALNs (mesenteric, paracolic, ileocecal lymph nodes), shown in blue, and NGALNs (hepatic hilar, common iliac, inguinal lymph nodes), shown in red. Created in BioRender. S, T. (2026) https://BioRender.com/hymu7db. (**B**) SIV DNA and RNA levels in GALNs and NGALNs, quantified by real-time PCR. (**C**) Histopathological and immunohistochemical assessment of GALNs and NGALNs using Masson’s trichrome staining (left), H&E staining (middle), and CD20 immunostaining (right). Yellow arrows indicate fibrosis and red arrows indicate B cell zones. (**D**) Multiplex immunofluorescence analysis of GALNs and NGALNs. Left panels: CD4 (red) and CD20 (green) co-staining. Right panels: CD8 (green) and CD20 (red) co-staining. Nuclei were counterstained with DAPI (blue). (**E**) Gene set enrichment analysis (GSEA) showing downregulated pathways in GALNs compared with NGALNs. Adjusted *p* < 0.05. (**F**) GSEA showing immune-related pathways differentially enriched between GALNs and NGALNs. Adjusted *p* < 0.05.

The rhesus macaque lacked protective MHC class I alleles associated with spontaneous control of SIVmac239 replication, including Mamu-A*01, Mamu-B*03, Mamu-B*17, Mamu-B*1702, and Mamu-B*08, with Mamu-DRB and Mamu-B detected as internal controls (Figure S1B). During the final year prior to necropsy, longitudinal monitoring revealed a progressive immunological deterioration: the CD4/CD8 ratio declined steadily from 0.6 to 0.22, while plasma viral loads fluctuated from approximately 2000 copies/mL, surged to 8000 copies/mL between months 6–9, and then declined to 1000 copies/mL at the terminal time point (Figure S1C,D). At necropsy, the animal had progressed to terminal AIDS, with persistent diarrhea for more than 2 weeks, severe weight loss, lethargy, plasma viral load of 1192 copies/mL, CD4+ T cell count of 30 cells/μL, CD8+ T cell count of 136 cells/μL, and a CD4/CD8 ratio of 0.22. Lymph nodes were classified as GALNs (mesenteric, paracolic, ileocecal lymph nodes) or NGALNs (hepatic hilar, common iliac, inguinal lymph nodes) (Figure 1A). Quantification of viral DNA and RNA loads revealed a marked anatomical shift in reservoir distribution at the terminal stage (Figure 1B). SIV DNA was substantially lower in GALNs than in NGALNs, whereas viral RNA was detected only in NGALNs, indicating that NGALNs, rather than GALNs, represented the dominant viral reservoir compartment during terminal AIDS.

Histopathological and immunofluorescence analyses identified profound structural divergence between GALNs and NGALNs (Figure 1C,D). Masson’s trichrome staining showed extensive fibrosis in the superficial cortex, corresponding to B cell zones, in GALNs, whereas NGALNs exhibited minimal fibrotic remodeling. H&E staining further revealed severe paracortical (T cell zone) destruction and extensive cellular loss in GALNs, in contrast to the relatively preserved architecture of NGALNs, despite swollen paracortical cells. CD20 immunostaining confirmed pronounced atrophy of B cell zones in GALNs compared with NGALNs, demonstrating more severe structural disruption in the gut-associated compartment. Multiplex immunofluorescence for CD4 and CD20 showed near-complete loss of CD4+ T cells in GALN B cell zones, where these cells largely correspond to follicular helper T cells (TFH), an important HIV/SIV reservoir cell population, and in the paracortex. In contrast, NGALNs retained detectable CD4+ T cells in both regions, although paracortical CD4+ T cells exhibited abnormal swelling. This profound depletion of CD4+ T cells in GALNs likely accounts for their loss of predominant viral reservoir status. CD8 and CD20 co-staining further revealed massive infiltration of CD8+ T cells into GALN B cell zones, a pattern not observed in NGALNs. This ectopic CD8+ T cell infiltration into B cell follicles is consistent with follicular injury and lymphoid tissue exhaustion.

Transcriptomic profiling further distinguished GALNs from NGALNs at the functional level. Differential expression analysis identified 174 upregulated and 39 downregulated genes in GALNs relative to NGALNs. Given the limited number of individual DEGs, GSEA was subsequently performed to capture coordinated pathway-level changes. Four major pathway categories were significantly downregulated in GALNs relative to NGALNs: DNA replication and repair, RNA processing and metabolism, chromatin and epigenetic regulation, and cell-cycle and checkpoint control (Figure 1E). Broad suppression of these core cellular processes suggests a state of profound tissue exhaustion in GALNs. Immune pathway analysis further showed activation of innate humoral immune pathways but marked downregulation of adaptive immune response pathways in GALNs (Figure 1F). This transcriptomic shift is consistent with CD4+ T cell depletion and architectural collapse in GALNs, supporting the interpretation that GALNs transition from a major reservoir compartment to a structurally damaged and functionally exhausted immune compartment during terminal AIDS.

## 4. Discussion

Virological compartmentalization between GALNs and NGALNs has been well characterized during acute and chronic SIV infection, but the reservoir status of GALNs during terminal AIDS remains poorly defined. After acute intravenous SIV challenge, quantitative DNA PCR and in situ hybridization showed that GALNs were a dominant site of early viral replication at day 7 post-infection compared with NGALNs and spleen, although viral distribution became more broadly dispersed across lymphoid tissues by day 14 [11]. Consistent with this pattern, Rabezanahary et al. demonstrated that, at peak viremia on days 11–14, GALNs represented the principal anatomical reservoir of SIV DNA, harboring 14.6-fold higher SIV DNA levels than peripheral lymph nodes [4]. This gut-associated dominance also persisted into chronic infection. Measurements of cell-associated SIV DNA, viral RNA, and replication-competent virus by quantitative viral outgrowth assay identified GALNs as the predominant reservoir compared with peripheral blood mononuclear cells (PBMCs) and NGALNs, even in SIV-infected macaques with complete plasma viral suppression and undetectable viremia [3]. GALNs have also been implicated as a major source of plasma viral rebound and an early site of viral seeding after cART interruption [11,12]. The terminal-stage case described here revealed apparent reversal of this established pattern, with GALNs showing near-undetectable SIV DNA and no detectable viral RNA, suggesting that the reservoir function normally enriched in this compartment can be preferentially lost at end-stage disease.

This anatomical shift in viral persistence is consistent with region-specific lymphoid injury. During acute SIV infection, GALNs undergo CD4+ T cell loss and proinflammatory cytokine release driven in part by caspase-1-mediated pyroptosis [13]. At the same time, this compartment develops a pro-fibrotic immune environment characterized by elevated Treg frequency, distorted Th17/Treg balance, and sustained expression of IDO-1, TGF-β1, and collagen-1 mRNA, changes that can persist despite early cART [14]. In chronic infection, GALNs contain substantially fewer CD4+ T cells than NGALNs and show increased CD8+ T cell activation [15]. Transcriptional studies further indicate that lymph nodes exhibit pronounced model-specific gene expression changes at peak viral load: pathogenic infection is associated with cellular stress, Th1 polarization, and sustained type I and II interferon responses, whereas nonpathogenic infection induces a strong but transient type I interferon response that resolves after peak viremia [16]. Similarly, progressive chronic infection is accompanied by increased expression of interferon-induced, inflammatory, and immune activation-related genes in GALNs, whereas long-term asymptomatic infection is characterized by suppressed viral replication, preserved CD4+ T cells, and downregulation of these pathways [17]. In the present terminal-stage case, GALNs showed severe fibrotic remodeling, paracortical destruction, CD4+ T cell depletion across follicular and paracortical regions, and extensive CD8+ T cell accumulation within B cell follicles. Transcriptomic profiling further revealed broad repression of DNA replication and repair, RNA processing, cell-cycle regulation, and checkpoint control, together with innate immune activation and attenuation of adaptive immune pathways—a signature consistent with tissue exhaustion, indicating that the same compartment undergoes preferential structural and functional collapse at terminal AIDS.

Terminal-stage GALNs displayed profound disruption of the B cell zone, with several coordinated features consistent with impaired humoral immunity. First, marked fibrosis and follicular atrophy disrupted B cell zone architecture, likely compromising GC organization and B cell survival [1,18]. Second, extensive TFH cell depletion indicated loss of essential GC support, with likely consequences for B cell maturation, antibody production, and antiviral humoral responses [19]. Third, extensive CD8+ T cell infiltration into B cell follicles further exacerbated follicular injury, as functionally exhausted CD8+ T cells have limited capacity to eliminate infected targets, whereas regulatory CD8+ T cell subsets suppress TFH-derived IL-21 production and B cell IgG responses [20,21]. Collectively, B cell zone fibrosis, TFH cell depletion, and intrafollicular CD8+ T cell accumulation indicate that the humoral immune niche in terminal-stage GALNs is structurally and functionally compromised, consistent with profound suppression of humoral immunity.

The T cell compartment in GALNs was also severely damaged. Profound depletion of CD4+ T cells in the paracortex and B cell follicles reduced the major cellular targets for productive HIV/SIV infection, providing a plausible explanation for the low SIV DNA burden and absence of detectable viral RNA in GALNs. This loss is also consistent with collapse of cellular immune capacity within the GALN compartment. Transcriptomic analysis corroborated these findings: compared with NGALNs, GALNs showed significant downregulation of adaptive immune pathways, including B cell activation, lymphocyte-mediated immunity, and antigen receptor signaling (adjusted *p* < 0.05). This molecular signature aligns with the histological evidence of CD4+ T cell depletion and architectural collapse, indicating coordinated failure of both humoral and cellular immunity in end-stage GALNs.

The preferential exhaustion of GALNs is highly relevant to gut barrier failure during advanced HIV/SIV disease. The gut is continuously exposed to microbial and dietary antigens and relies on both epithelial integrity and gut-associated lymphoid tissues, including GALNs, to maintain immune containment. During HIV/SIV infection, viral replication disrupts epithelial barriers and depletes CD4+ T cells across mucosal, submucosal, and lymphoid compartments, thereby promoting microbial translocation into the circulation and driving persistent systemic immune activation that fuels disease progression [22,23]. In this terminal-stage macaque, severe GALN exhaustion reflects failure of this gut-associated immunological barrier. Upregulation of antimicrobial humoral immune pathways in GALNs also suggests ongoing microbial exposure or invasion within this compartment. These observations support the interpretation that exhaustion of GALNs, together with loss of gut barrier integrity, may allow persistent microbial translocation to drive systemic immunopathology, thereby accelerating progression toward terminal AIDS.

Several limitations should be considered. First, this was a single-animal case report; therefore, interindividual variation cannot be excluded, and validation in additional terminal-stage SIV models is required. Second, the animal did not receive antiretroviral therapy, which differs from most clinical reservoir studies conducted under cART. Nevertheless, untreated infection provides a direct view of terminal AIDS pathology without pharmacological suppression of viral replication. Third, bulk RNA-seq cannot resolve the specific cell populations responsible for the observed exhaustion signature. As such, single-cell and spatial transcriptomic approaches will be needed to define the cellular sources and anatomical organization of these transcriptional changes.

In conclusion, this case indicates that long-term SIV infection can drive preferential structural and functional exhaustion of GALNs during terminal AIDS, leading to the loss of predominant viral reservoir status relative to NGALNs. These findings reveal that lymph node compartments at different anatomical sites follow distinct pathological trajectories during advanced HIV/SIV infection and highlight gut-associated immune barrier collapse as a central feature of progression to terminal AIDS.

## Data Availability

Sequencing data supporting the findings of this study have been deposited in the Genome Sequence Archive (GSA) database system under accession code CRA044488 (Shared URL: https://ngdc.cncb.ac.cn/gsa/s/T2ncVgfM (accessed on 9 June 2026)).

## Supplementary Materials

The following supporting information can be downloaded at: https://www.mdpi.com/article/10.3390/v18080855/s1, Figure S1: MHC class I/II genotyping and longitudinal virological and immunological parameters during the final year prior to necropsy.

## Abbreviations

The following abbreviations are used in this manuscript:

AIDS	Acquired immunodeficiency syndrome
cART	Combination antiretroviral therapy
DAPI	4′,6-diamidino-2-phenylindole
DEGs	Differentially expressed genes
GALNs	Gut-associated lymph nodes
GCs	Germinal centers
GSEA	Gene set enrichment analysis
H&E	Hematoxylin–eosin
HIV	Human immunodeficiency virus
NGALNs	Non-gut-associated lymph nodes
PCR	Polymerase chain reaction
PCR-SSP	Polymerase chain reaction with sequence-specific primers
PBS	Phosphate-buffered saline
RNA-seq	RNA-sequencing
SIV	Simian immunodeficiency virus
TFH	Follicular helper T cells
