# Supplementary material for: Preferential Exhaustion and Loss of Predominant Reservoir in Gut-Associated Lymph Nodes of a SIVmac239-Infected Rhesus Macaque with Terminal AIDS
Source: Viruses. 2026 Aug 4;18(8):855. doi: 10.3390/v18080855 (PMC13517617; doi:10.3390/v18080855)
Supplement: Supplementary file 1 [file viruses-18-00855-s001.zip › viruses-4394529-supplementary.pdf]

A

MHC class I/II target	Forward	Reverse
Mamu-A*01	GACAGCGACGCCGCGAGCCAA	GCTGCAGCGTCTCCTTCCCC
Mamu-B*08	CGTGAGGCGGAGCAGGTC	CCACAGCTCCGATGAACACAG
Mamu-B*03	TTCGTGCGGTTCGACAGT	GTTCCATCTCCTCCTGGCCTA
Mamu-B*17	GCCGGCTCGCACTCCATGAA	GCGCGCTGCAGCGTCTCC
Mamu-B*1702	AACGGTGACCTGCGCTCT	CTCTTCTCCAGAAGGCACCAA
Mamu-B	AGAGCAGCGGAGAGCCTACCT	GCTGCACATGGCACGTGTATC
Mamu-DRB	GCCTCGAGTGTCCCCCCAGCACGTTTC	GCCGCAGCTTTCACCTCGCCGCTG

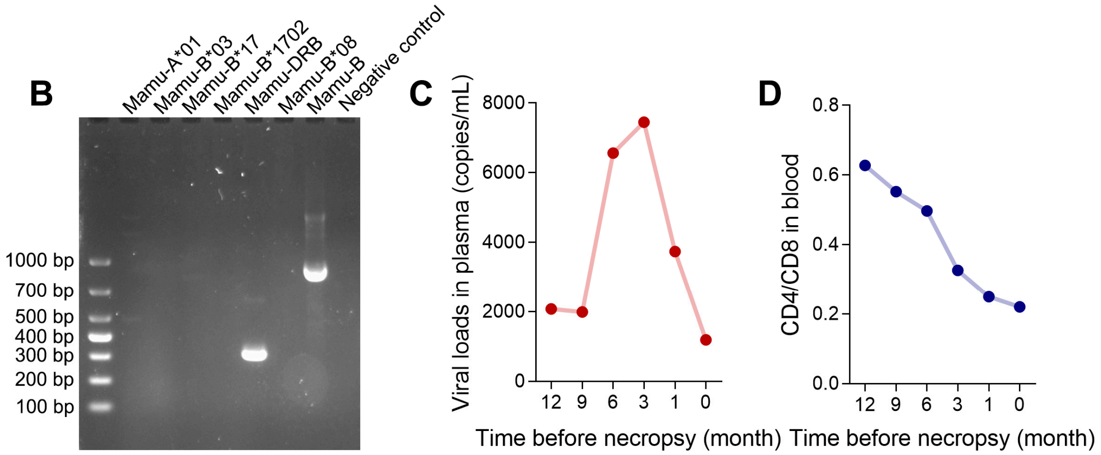

Figure S1. MHC class I/II genotyping and longitudinal virological and immunological parameters during the final year prior to necropsy. **(A)** Primer sequences used for MHC class I/II genotyping. **(B)** PCR-SSP results showing the absence of protective MHC class I alleles (Mamu-A*01, Mamu-B*03, Mamu-B*08, Mamu-B*17, and Mamu-B*1702), with Mamu-DRB and Mamu-B as internal controls. **(C)** Longitudinal plasma viral loads measured during the final year prior to necropsy. **(D)** Longitudinal CD4/CD8 ratios measured during the final year prior to necropsy.
